# Dartagnan: Leveraging Compiler Optimizations and the Price of Precision (Competition Contribution)

**DOI:** 10.1007/978-3-030-72013-1_26

**Published:** 2021-02-26

**Authors:** Hernán Ponce-de-León, Thomas Haas, Roland Meyer

**Affiliations:** 8 Eindhoven University of Technology, Eindhoven, The Netherlands; 9 Aalborg University, Aalborg East, Denmark; 10 Bundeswehr University Munich, Munich, Germany; 11 TU Braunschweig, Braunschweig, Germany

## Abstract

We describe the new features of the bounded model checker Dartagnan for SV-COMP ’21. We participate, for the first time, in the *ReachSafety* category on the verification of sequential programs. In some of these verification tasks, bugs only show up after many loop iterations, which is a challenge for bounded model checking. We address the challenge by simplifying the structure of the input program while preserving its semantics. For simplification, we leverage common compiler optimizations, which we get for free by using LLVM. Yet, there is a price to pay. Compiler optimizations may introduce bitwise operations, which require bit-precise reasoning. We evaluated an SMT encoding based on the theory of integers + bit conversions against one based on the theory of bit-vectors and found that the latter yields better performance. Compared to the unoptimized version of Dartagnan, the combination of compiler optimizations and bit-vectors yields a speed-up of an order of magnitude on average.

## 1 Overview

DARTAGNAN is a bounded model checking (BMC) tool for reachability analysis. It takes a program and converts it to an SMT formula representing all its executions up to a given bound. This formula, together with a reachability condition representing assertions, is passed to an SMT solver (we use Z3 as a backend). If the formula is satisfiable, an execution violating an assertion exists.

DARTAGNAN was initially developed to verify small concurrent programs (written in the `.litmus` format) under weak memory models. Since 2020, it also supports Boogie *intermediate verification language* as its input language. For C programs, we use SMACK [8] to compile to LLVM and transform the compiled code to Boogie. DARTAGNAN's architecture, and main verification techniques (in particular how to efficiently handle different memory models) are described in [3,4,7]. Version 2.0.7 participating in SV-COMP'21 [1] can be downloaded from https://github.com/hernanponcedeleon/Dat3M directly as a java archive (`.jar`) or built from source code using the Maven build system. DARTAGNAN's

* Jury member.

```
int main(void) {
  unsigned int x = 1;
  unsigned int y = 0; while (y < 1024) {
    x = 0;
    y++;
  }

__VERIFIER_assert(x == 0);
}
```

**Fig. 1.** Benchmark `const_1-1.c` from the *ReachSafety-Loop* category.

verifier archive to reproduce the results of SV-COMP'21 is published at Zenodo under DOI 10.5281/zenodo.4483224.

Last year Dartagnan only participated in the *ConcurrencySafety* category. What is new for SV-COMP'21 is that Dartagnan also participates in (part of) the *ReachSafety* category for single threaded programs. Many tasks in that category contain loops of large bounds which impacts Dartagnan's performance. To address the problem, we propose to leverage compiler optimizations.

## 2 Leveraging Compiler Optimizations

BMC techniques are very sensitive to the program syntax. The loop structure and the number of variables directly impact the size of the SMT formula (which tends to relate to solving times). Our approach is to simplify the structure of the program (while preserving its semantics) before performing the verification. We do this by using compiler optimizations.

Consider the program in Fig. 1 from the *ReachSafety-Loop* category. A BMC tool has to unroll the program 1024 times to prove the program correct. However, since the value of `x` is constant at every loop iteration, the assignment can be moved outside the loop. Since the value of `y` is never read, the instruction `y++` can be removed (using dead store elimination) leading to an empty loop which can also be removed. Finally, using constant propagation, the assertion can be re-written as `__VERIFIER_assert(0 == 0)` which is trivially true.

All these optimizations are implemented in most optimizing compilers. Since we perform the verification after compiling to LLVM, we get them for free. Due to the high number of loop iterations, Dartagnan needs more than 15 minutes to verify the program above. However, by using the `-O3` optimization flag in the C-to-Boogie transformation, the verification task can be solved within seconds.

Using an optimizing compiler has its risks. Most optimizations are unsound for concurrent programs [9] and we do not use any for *ConcurrencySafety*. Even for sequential programs, there is a price to pay. Some optimizations introduce bitwise operations (e.g. multiplications tend to be compiled to shift operations)

which were not present in the original program. We thus have to encode the semantics of such operations precisely.

## 3 The Price of Precision

To guarantee soundness when using the aforementioned compiler optimizations in the *ReachSafety* category, we use two precise encodings of integers. The first is a new implementation based on the theory of bit-vectors, where we get bit-precise reasoning for free. The second was our original implementation and it is based on the theory of integers. It does an *on-demand* conversion to bit-vectors and back (`Int2Bv` and `Bv2Int`). We are able to solve more benchmarks with the theory of bit-vectors than with the theory of integers plus conversion, which suggests that converting between the theories is expensive. For concurrent programs, the combination of bit-vectors with Dartagnan's memory-model-dependent encoding significantly degrades performance, and we use the theory of integers throughout the *ConcurrencySafety* category.

The trade-off between the efficiency of a theory and the precision in modeling semantics is well-known. In the context of symbolic execution, it was explored in [6]. SMACK implements an approach to diagnose spurious counterexamples caused by over-approximations and gradually refines the precision of reasoning about bitwise operations [5].

## 4 Evaluation

We evaluated how compiler optimizations and different integer encodings affect Dartagnan's verification capabilities for some benchmarks in the *ReachSafety* category. We support two levels of optimization: `-O0` (no optimization) and `-O3` (enables most optimizations). For integer encodings we use two different approaches: theory of integers + bit conversions (`QF_LIA` + `QF_BV` logics) and pure theory of bit-vectors (`QF_BV` logic).

The results are given in Fig. 2. We use Benchexec [2] for reliable benchmarking. The graph shows the verification time w.r.t the verification score. Following the competition scheme, correct counter-examples and proofs give +1 and +2 points respectively. Wrong counter-examples and proofs give -16 and -32 points. The absolute score values for incorrect results are higher because a single correct answer should not compensate for a wrong answer.

It can be seen that, regardless of the chosen integer encoding, using compiler optimizations allows us to verify many more benchmarks, thus obtaining a higher score. The total number of solved tasks with no optimizations (`O0+Bit-vectors` and `O0+Int-exact` configurations from Fig. 2) is 89 with 77 correct and 12 incorrect results. When using optimizations (`O3+Bit-vectors` and `O3+Int-exact` configurations), we solved 336 tasks with 326 correct and 10 incorrect results.

The experiments show that combining theories to achieve precision is more expensive than using pure bit-vectors. The total number of solved tasks when using `QF_LIA` + `QF_BV` (configurations `O0+Int-exact` and `O3+Int-exact`) is 201

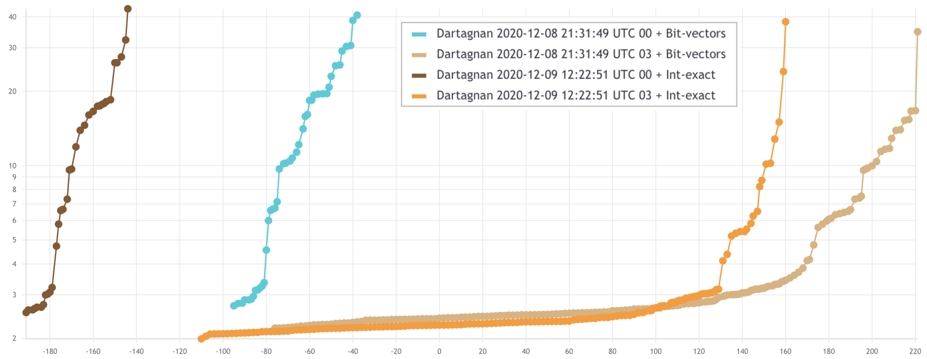

**Fig. 2.** Comparing the performance of Dartagnan with different optimization flags and integer encodings.

with 187 correct and 14 incorrect results. When using `QF_BV` (configurations `O0+Bit-vectors` and `O3+Bit-vectors`) we solved 224 tasks with 216 correct and 8 incorrect results. All encodings are guaranteed to be sound, the incorrect results are due to bugs in the verifier.

We used the evaluation described above to decide the configuration for SV-COMP'21. For category *ConcurrencySafety*, we use the integer encoding and no compiler optimizations. For categories *ReachSafety-Loop*, *ReachSafety-BitVectors* and *ReachSafety-Arrays*, Dartagnan uses the theory of bit-vectors and `-O3` optimizations. These configurations are internally decided by the tool based on the use of the pthreads library. Compared with SV-COMP'20, we solved 60 more tasks in *ConcurrencySafety* (55% increase) and 474 more tasks overall (582% increase).

***Acknowledgement:*** We thank the SMACK developers for their constant support with the C-to-Boogie transformation. We also thank Yun Zhang for her contributions to the development of the witness generation.
